# Surgeon Moyle Breton

**Published:** 1910-07-30

**Authors:** 


Invalided from the Navy Medical Service in consequence of an illness of some years' duration, Surgeon Moyle Breton died last week at a nursing home at Guildford, at the comparatively early age of forty-one. He was a student of St. George's Hospital, and represented the hospital at the Inter-Hospital Sports at Stamford Bridge in 1895. He took his M.R.C.S., L.R.C.P. in 1895, and two years later entered the Navy and was appointed a surgeon to the *Victory* at Portsmouth in 1904.

